# Gaze Tracking of Pitched Balls in Coupled and Uncoupled Tasks

**DOI:** 10.3390/vision10030051

**Published:** 2026-08-06

**Authors:** Mason Clutter, Nick Fogt

**Affiliations:** College of Optometry, The Ohio State University, 338 West Tenth Avenue, Columbus, OH 43210, USA; clutter.50@osu.edu

**Keywords:** eye movement, sports, perception-action coupling, baseball, batting

## Abstract

Studies suggest that anticipation in sports-related tasks is better if the task maintains the coupling of perception and action required in competition. Study 1 addressed the question of whether gaze tracking varies between coupled tasks in baseball and softball batters. Fifteen subjects executed either a coupled task (partially swinging a bat at an approaching ball) or an uncoupled task (predicting the passing height of an approaching ball). The ball was stopped by a net 8 feet (2.44 m) in front of the subject. Rapid gaze shifts occurred for 53.0% of the pitches in the uncoupled task, and for 39.7% of the pitches in the coupled task (*p* = 0.039). The number of predictive gaze shifts toward the terminal ball location in each condition (i.e., the expected location of bat–ball contact in the coupled condition, and the observer in the uncoupled condition) did not vary between the conditions (*p* = 0.388). Study 2 examined whether gaze fixations on the net found in Study 1 occurred because passing height judgments could not be improved at distances closer than 8 feet from the observer. Passing height judgments were lower when the ball was allowed to pass by the observer, suggesting that subjects processed trajectory information within 8 feet.

## 1. Introduction

Baseball batting is one of the most visually demanding tasks in sports. Fastballs can regularly reach over 90 miles per hour (145 kilometers per hour) at adult levels of play, such that the ball travels from the pitcher’s hand to the batter in less than 400 ms. Further, the bat swing generally takes 150–200 milliseconds, leaving very little time for the batter to decide whether and how to swing the bat [1,2,3].

In the sports literature, a differentiation between coupled and uncoupled tasks has been made. In a coupled task, perception and action are coupled as in an actual competition or game, while in an uncoupled task perception and action are not coupled, so the required action is different from that required in an actual competition or game [4,5,6,7,8]. In baseball batting, for example, a coupled task would be swinging a bat at pitched balls while an uncoupled task would be judging the height upon arrival of a pitch. The question of whether anticipatory behaviors vary between coupled and uncoupled tasks is an important one, because limitations on training space and training time may restrict the use of coupled tasks in training athletes.

In comparing anticipatory performance with coupled and uncoupled tasks, Farrow and Abernethy conducted experiments on tennis players [4]. The subject’s task was to make a prediction of the direction of an opponent’s serve. In some cases, these predictions were assessed by examining the direction in which subjects moved as they emulated returning the serve (coupled response). In other cases, subjects simply made a verbal prediction of the serve direction (uncoupled response). Anticipatory performance was found to be better in the coupled task when a portion of the ball’s flight was visible to subjects. Similarly, better predictive performance in coupled conditions compared to uncoupled conditions was reported by Mann and colleagues in cricket batters [7]. In contrast, Ranganathan and Carlton performed a baseball study in which subjects had to either verbally indicate whether a pitch was a fastball or a change-up in baseball in a virtual setting (representation of a baseball pitcher projected onto a screen) [5]. What these latter investigators reported was opposite to the findings of Farrow and Abernethy [4]. Baseball batters were significantly more accurate at predicting which pitch type had been thrown in the verbal response (uncoupled) condition compared to the bat swing (coupled) condition. This was true even when ball-flight information was unavailable. Ranganathan and Carlton suggested that their findings deviated from those of Farrow and Abernethy because their baseball batting task was more difficult than the coupled task used by Farrow and Abernethy (i.e., moving in the direction of a tennis serve), and because baseball batters have more time to make their anticipatory decisions when assessing the pitch type verbally (the uncoupled task) compared to the time available for a bat swing (the coupled task). It has been suggested that Ranganathan and Carlton’s findings resulted from the fact that the point-light display they used was unnatural and could not be considered coupled [7,8].

Based at least partially on the differences in coupled and uncoupled performance in the just-described studies, many investigators have put forward the argument that in order to properly assess the anticipatory responses of athletes, studies of these responses should be made in coupled environments that are representative of the situations and tasks encountered in actual competitions [9,10,11].

While studies have shown differences in predictive responses for coupled and uncoupled tasks, a question that remains is whether gaze behavior varies for these tasks. This is the question to be addressed in this experiment. Throughout this paper, the term gaze will refer to the location of the eyes in the visual scene as determined by combining eye-in-head and head rotations. An understanding of the nature of the gaze movements employed in sports-related tasks can inform investigators on whether and how these movements influence visuomotor performance [12]. At least one group has compared ocular fixations in coupled and uncoupled conditions. Dicks and colleagues looked at fixations of football (soccer) goalkeepers from the New Zealand Southern Premier League who were facing penalty kicks on video and in situ. In the in situ conditions, goalkeepers responded in one of three ways [6]. The response was either a verbal indication of the direction of the penalty kick, or a “side step” with placement of the arms in the anticipated direction of the kick, or a naturalistic movement in an attempt to intercept the kick. The goalkeepers were more likely to fixate on the ball early in the course of the penalty kicker’s run-up when they attempted to intercept the kicks, compared to when they made a verbal or partial interceptive response. This suggests that ocular fixation behaviors vary between coupled and uncoupled tasks.

Different from the study of Dicks and colleagues, the study to be described in this paper examines gaze behaviors in response to an approaching ball. Eye and head movements associated with interception of approaching balls have been studied in a number of contexts [13,14,15,16,17,18,19,20,21,22]. The picture that emerges is that after a period of gaze tracking, individuals often make predictive gaze shifts (typically predictive saccades or combined eye and head gaze shifts) to a predicted spatial location at which interception or contact with the approaching object is expected to occur [13,14,15,16,17,19,20,21,22]. Alternatively, in cases where the ball is expected to bounce, predictive gaze shifts may place the gaze at a location that is expected to be occupied by the ball after it bounces [16,19]. Once gaze is placed at one of these predicted locations, then a predictive fixation occurs in which the gaze “lies in wait” for the ball to arrive [14,15,16,19,20,21,22]. This predictive fixation may allow for early “encoding” of object location to facilitate the motor plan involved in intercepting the object, or this fixation might improve future predictions of interception location, or this fixation may facilitate tracking of the object after it bounces [14,15,16,19,20]. As a result, these predictive fixations may improve the spatial and temporal accuracy of visuomotor responses [21].

In this paper, gaze movements with anticipatory performance associated with pitched baseballs will be compared in coupled and uncoupled tasks. Recent studies of baseball batters suggest that both the eyes and head may be involved in tracking pitched balls, and some studies suggest that predictive saccades to the predicted location of contact between the bat and ball can occur [2,17,18,20,23]. Some of these studies required batters to simply track the ball while in other studies batters swung a bat at the balls. However, there are little data on whether the gaze movements of baseball batters vary depending on whether the task is coupled (bat swing) or uncoupled (no bat swing). Fogt and Persson are the only investigators to directly address the question of whether the baseball batter’s task influences eye and head movements [17,23]. In one published study, they reported on horizontal eye and head movements in two former collegiate baseball players viewing tennis balls thrown from a pneumatic pitching machine [17]. There were two conditions: in the coupled condition subjects swung a bat at the approaching ball, and in the uncoupled conditions subjects “took” the pitch. That is, in the uncoupled condition observers simply observed the ball as it passed by them. Predictive gaze shifts ahead of the ball occurred when batters observed pitches, while continuous gaze tracking occurred when batters swung at pitches. It was suggested that the predictive gaze shifts in the uncoupled condition may allow batters to compare the predicted trajectory of the ball to the actual arrival location, which may aid in future swing decisions [2,17,18,19,20]. Continuous tracking in the coupled or bat swing condition may increase the likelihood of successful bat–ball contact, as extraretinal signals associated with the motor commands sent to the head musculature and the extraocular muscles may be useful in predicting where and when the approaching ball will arrive [2,24]. Additionally, tracking the ball continuously provides feedback signals from retinal images of the ball and the surrounding environment.

While the results from Fogt and Persson suggest that there may be differences in gaze behavior in coupled and uncoupled tasks related to baseball batting, there are no data comparing these gaze behaviors when an actual judgment of ball location is required in the uncoupled task. Therefore, the primary purpose of the first study described here was to compare gaze movements of baseball and softball batters in a coupled task (partially swinging a bat at an approaching ball) and an uncoupled task (judging the vertical passing height of an approaching ball). Passing height as defined here is the vertical height from the ground at the time the ball passes by the observer. For both the coupled and uncoupled tasks, the pitched ball was stopped by a net placed between the subjects and the pitching machine used to “throw” the approaching ball. The hypothesis was that the number of predictive gaze shifts in the direction of the location where the task was terminated (the plate adjacent to the observer in the uncoupled condition and the expected location of bat–ball contact in the coupled condition) would be greater in the uncoupled condition when judgments of passing height were required. In the uncoupled or judgment condition, predictive gaze shifts from the last point in the ball’s trajectory (i.e., when the ball struck the net) in the direction of the plate adjacent to which the batter stood, followed by a predictive fixation, would help to ensure that the ongoing or online estimation of the ball’s trajectory as it approached could be mapped onto the vertical meter stick placed adjacent to the subjects [20,21,22,25,26]. In the coupled (partial swing) condition, the decision on where and when to swing the bat must be completed early in the trajectory, so it was hypothesized that predictive gaze shifts and subsequent predictive fixations were less likely to occur in that condition as their benefit is diminished. To address some questions generated by the first study, a second study was completed as described below.

## 2. Studies 1 and 2

### 2.1. Study 1

#### 2.1.1. Materials and Methods—Study 1

For Study 1, the protocol (IRB protocol #2023H0212) and the associated consent forms were approved by the Ohio State University Biomedical Sciences Institutional Review Board. Subjects signed an informed consent form prior to participation in the study. Subjects were recruited using the Study Search website administered through The Ohio State University Center for Clinical and Translational Science, as well as through an e-mailed study advertisement to The Ohio State University College of Optometry staff, faculty, and students.

Fifteen subjects participated in this study (5 females), mean age 24.6 years old (range 22–27). In order to be eligible for the study, subjects were required to be 18 to 40 years of age, to have 20/20 best corrected monocular visual acuity in both eyes, to demonstrate no strabismus/heterotropia upon cover test in primary gaze as well as left and right gaze, and to have stereoacuity of at least 60 s of arc on a Randot stereo acuity test. For two subjects, the presence of strabismus was only assessed in primary gaze. Subjects were required to have played baseball or softball at the high school level or above within the last 10 years.

Subjects were given a short questionnaire to fill out assessing their level of experience in baseball and softball. The three questions in the questionnaire were as follows:

1. Which sport (baseball or softball) did you (or do you) play?
2. Were you primarily a pitcher, a batter, or both and/or did this role change over your playing years (that is, did your primary position change at the highest levels versus the lower levels at which you played)?
3. What is the highest level at which you played (or play) this sport?

Based on the survey responses, all subjects were either primarily batters or both a pitcher and a batter. Excluding recreational play in college, the highest level at which 9 subjects played was high school. The other 6 subjects reported that they played at the collegiate level.

Tennis balls were pitched to subjects using a pneumatic pitching machine (Flamethrower, Accelerated Baseball Technologies, Crystal Lake, IL, USA) [17,23]. The balls were fed into the pitching machine one at a time by one of the investigators. The balls exited the pitching machine through an attached PVC pipe held in place by a tripod. The pitching machine and the speed adjustment on the PVC pipe was situated behind a dark hanging shroud such that subjects could not see the investigator adjust the pitching speed between pitches.

Two speed settings were used for this study. Every tenth pitch was thrown at the second, slower speed to allow for some variation in pitch trajectory. The speed of the pitch could be modulated by covering or exposing holes on the PVC pipe from which the balls were thrown using a sliding sleeve around the tube. For the two pitch speed settings, the time required to traverse distances from the pitching machine of 10 feet (3.05 m), 20 feet (6.10 m), 30 feet (9.14 m), and 40 feet (12.19 m) were assessed 10 times using two timing windows as described previously [17]. These values were then converted to (mean) velocities at that distance. The results are shown in Table 1 below.

**Figure 1:**
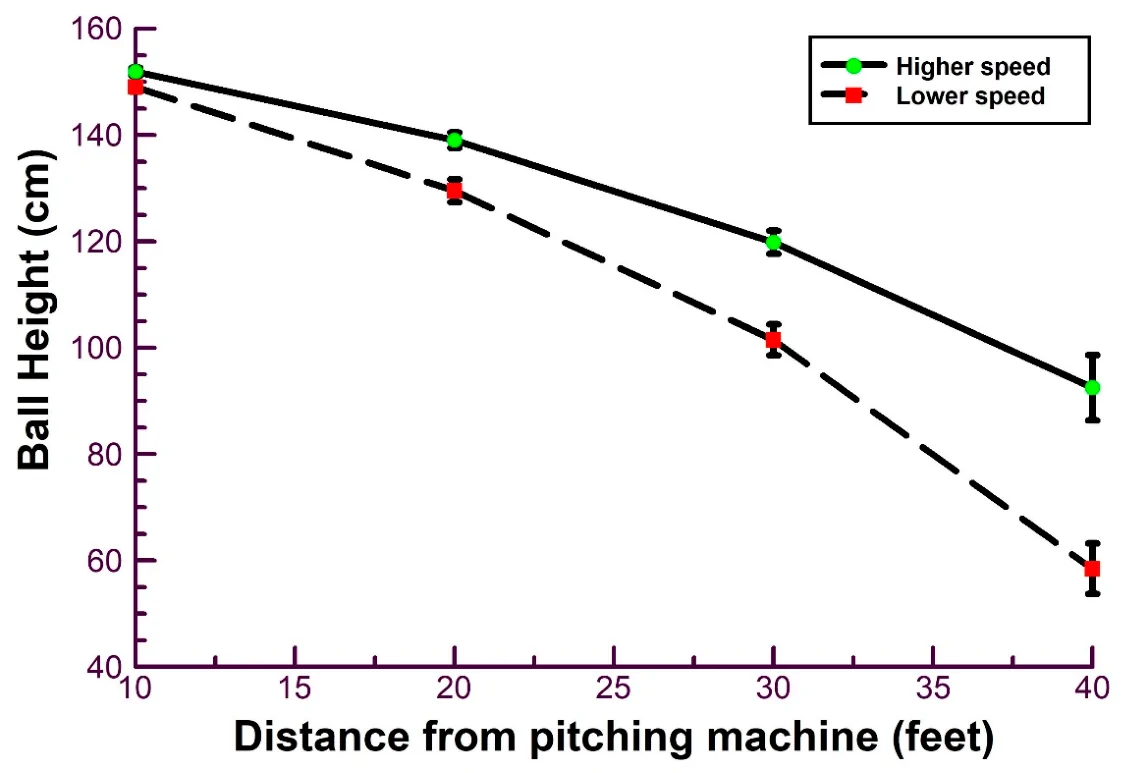
Mean pitch heights (plus/minus the standard deviation) at different distances from the pitching machine.

The height of the ball upon arrival at each of the four distances at which the ball speed was assessed was also measured 10 times as described previously [17,23]. The results of these measurements are shown in Figure 1.

The subject’s gaze was tracked using a monocular Pupil Core Eye Tracker (Pupil Labs GmbH, Berlin, Germany). The system recorded the location of the right eye (120 Hz) along with the scene (30 Hz) viewed by the subject. The system produced a video from the scene camera with a circular marker showing the location of gaze (eye-in-head rotation + head rotation) in the scene. It was possible to obtain numerical data representing the eye-in-head rotation, although the analyses described in this paper were based on the videos from the scene camera.

To calibrate the eye tracker, five fixation points were used. The calibration procedure utilized the “Natural Feature” calibration choreography included with the eye tracking software (Pupil Capture). Fixation targets were placed on two thin vertical posts and two concrete support columns. The two posts were placed in the path of the ball at 20 feet (6.10 m) and at 30 feet (9.14 m) from the end of the pitching machine tube. The fixation markers on these two posts were placed at the average expected height of the ball during the ball’s approach, as assessed from the height measurements described above. The fixation markers on the two support columns were about 9.3 deg to the left of the ball’s path (column 10 feet (3.05 m) from the end of the pitching machine tube) and 13.6 deg to the right of the ball’s path (column about 30 feet (9.14 m) from the end of the pitching machine tube). The fifth calibration target was the end of the pipe from which the ball was ejected from the pitching machine.

During the calibration, the subject stood at a distance of 40 feet (12.19 m) from the pitching machine. They were instructed to keep their head as still as possible, and to move only their eyes to fixate each of the calibration points. Immediately following the calibration, the subject was directed to look through all the calibration points once more while the investigator ensured accurate gaze tracking, in that the gaze location matched within about 1.25 deg (half the diameter of the circular eye location indicator) for each of the original fixation targets. To verify the calibration, the investigator viewed the video feed from the eye tracking scene camera and the gaze position indicator overlaid on this scene. If the calibration was deemed inadequate, meaning that gaze was more than 1.25 deg from any fixation target, it was performed again. Subjects were then allowed to move their heads as normal during the trials. Just prior to the experimental trials, the subject was instructed to look at the opening of the PVC pipe from which the ball exited the pitching machine as a final check on the calibration. Since the location of the eyes when the pitches were first released from the pitching machine did not vary significantly throughout the experimental trials, the calibration was thought to be stable throughout each trial.

A “plate” was placed on the ground 40 feet (12.19 m) away from the pitching machine. The subject was instructed to stand either to the left or right of this plate according to their hand dominance. A net was placed approximately 8 feet (2.44 m) away from the subject to stop the ball prior to it reaching the subject. In stopping the balls with the net, subjects were presumably required to extrapolate the ball’s trajectory over the distance from the net to the plate to estimate the ball’s passing height had the ball arrived at the plate.

Before the pitches for data collection were thrown, the net was placed 2 feet (0.61 m) in front of the subject, and one pitch at each of the two speeds used in the experiment was thrown to familiarize the subject with the general speed and height range of the pitches. After the net was once again placed 8 feet (2.44 m) in front of the plate, two randomized conditions were performed. Eye-in-head movements and gaze location relative to the scene were recorded throughout these trials with the Pupil Labs eye tracker. In one condition (the uncoupled condition hereafter referred to as the “predictive” condition), thirty pitches were thrown, and the subject was asked to observe the ball and estimate the height of the ball had the ball passed by them. A two-meter ruler (referred to as the “meter stick”) was placed beside the plate opposite the subject to quantify the height assessments. The subject reported their passing height estimate to the experimenter who recorded these responses in an Excel spreadsheet.

In the other condition, hereafter referred to as the “swing” condition, the subject was given a youth-sized wooden bat and asked to swing the bat at thirty pitches as if they were going to hit the ball. This was considered the coupled condition. Subjects were instructed to make a partial swing rather than a full swing because of space restrictions in the laboratory.

For each of the fifteen subjects, 20 pitches were analyzed for each of the two conditions, such that 600 pitches in total were analyzed. The experimenters watched the video from each pitch frame-by-frame at 30 Hz in the Pupil Player software provided by Pupil Labs, and documented the presence of rapid gaze shifts, which were evident as multiple gaze locations captured during the 33 ms (30 Hz) exposure duration of each camera frame. Slower gaze movements would often demonstrate just one gaze location in a frame or two closely spaced gaze locations. The analyses described here refer to rapid gaze shifts rather than pursuit eye movements or saccadic eye movements because the eye location from the scene camera was derived from the combination of eye-in-head and head rotations (i.e., the eye location indicator showed the gaze location rather than just the eye-in-head rotation). A determination was also made regarding whether rapid gaze shifts were directed toward the plate when passing height estimates were made or were in the direction of bat–ball contact when the baseball bat was swung. All statistical tests for Study 1 and Study 2 were completed in SPSS v.31 (IBM Corporation, New York, NY, USA) and Minitab v. 22 (Minitab LLC, State College, PA, USA).

#### 2.1.2. Results—Study 1

For Study 1, the first 20 pitches of each condition were analyzed for 13 subjects. For the other 2 subjects, 20 pitches were analyzed, but not all of the pitches were in the first 20. For one subject, this was because the first 10 pitches occurred with the meter stick on top of the plate and the meter stick was subsequently moved to the opposite batter’s box in order to increase the amount of the meter stick viewed by the eye tracker scene camera. For the other subject, the batter did not swing the bat at two of the first 20 pitches, so those videos for the 2 pitches for which no swing occurred were excluded and the videos associated with the 21st and 22nd pitches were added to the analysis.

One author (M.C.) initially performed the analyses. The second investigator (N.F.) repeated the analyses. The differences in the number of rapid gaze shifts for the predictive and swing conditions for each subject were calculated from each author’s analysis, and then an intraclass correlation coefficient (ICC) for these difference values was used to assess the inter-rater reliability of the procedure to recognize rapid gaze shifts. The ICC for the two investigators was 0.909 (95% confidence interval: 0.736–0.969) demonstrating good inter-rater reliability.

Because of the relatively low recording rate of the scene camera, it is possible that smaller gaze shifts, less than about 5 deg, could have been missed or mischaracterized as following movements rather than rapid gaze shifts. For example, a saccade of 2 deg is expected to require about 30 ms or less and could therefore be missed entirely or its trajectory may only be partially captured [27]. As mentioned, the circular eye location indicator was set at about 2.5 deg of visual angle relative to the visual scene at 40 feet in the Pupil Core video viewing software (Pupil Player, Pupil Labs GmbH, Berlin, Germany), providing a tool to assess the amplitude of rapid gaze shifts. The 2.5 deg setting for the location indicator was intended to partially account for the limitations imposed by the sampling rate of the scene camera, as apparent rapid gaze shifts less than 2.5 deg could not be reliably classified as rapid gaze shifts.

Periods of continuous or near continuous (i.e., the tracking may have consisted of both smooth periods of tracking along with rapid gaze shifts to the ball) tracking were common prior to the time the ball struck the net, although for some subjects there were conditions where continuous or near continuous was typically absent. For 2 subjects, poor continuous or poor near continuous tracking occurred in both the predictive condition and the swing condition. For 3 subjects, poor continuous or poor near continuous tracking occurred only in the swing condition. Rapid gaze shifts occurred in 53.0% of the trials in the predictive condition (mean and standard deviation = 10.6 ± 7.11, median = 11), and 39.7% (mean and standard deviation = 7.93 ± 6.69, median = 10) in the swing condition. The difference in the number of rapid gaze shifts between the two conditions was significant (Wilcoxon signed rank test: median difference = 2.5, 95% confidence interval: (0, 5.5), *p* = 0.039). Among the 15 subjects, 3 subjects made more rapid gaze shifts in the swing condition than in the predictive condition, 10 subjects made more rapid gaze shifts in the predictive condition than in the swing condition, and 2 subjects made equal numbers of rapid gaze shifts in the two conditions (including one subject who made no rapid gaze shifts in either condition).

The rapid gaze shifts of most interest in this study were those that could be considered predictive gaze shifts. Two categories of predictive gaze shifts were documented. One of these categories included those pitches where gaze was shifted past the ball towards the plate prior to the time the ball struck the net. To be included in this category, the ball had to lag behind the gaze location following the gaze shift by at least 1.25 deg (half the diameter of the gaze position indicator in the scene) and the landing location of the gaze shift had to be on the side of the net opposite the observer (that is, on the pitching machine side of the net). All of the pitches in which a rapid gaze shift was originally found were examined. In the predictive condition, there were only 12 examples of these predictive gaze shifts. In the swing condition, there were only 7 examples of these gaze shifts. Because the number of predictive gaze shifts in both conditions was very low, no further analyses of these data was completed.

The second category of predictive gaze shifts was most directly related to the study hypotheses. To be placed in this category, rapid gaze shifts must have started prior to or at the same time (i.e., in the same video frame) that the ball struck the net. The predictive gaze shift then had to continue past the net in the direction of the meter stick in the predictive condition or had to continue past the net in the direction of expected bat–ball contact in the swing condition. Rapid gaze shifts that were preceded by a gaze fixation at or near the net were not included in this category of predictive gaze shifts for the following reasons.

When gaze fixation remained at the net, the period of fixation was sometimes very short (about 2 video frames), although these fixations typically occurred over about 5–10 video frames (167–333 ms). Following these fixations, in the predictive condition the gaze was shifted rapidly in the direction of or to the meter stick at the plate. Some of these fixation periods on the net exceeded the time required for the ball to traverse the last 10 feet (3.05 m) of the pitch trajectory (about 110 ms). Because those rapid gaze shifts that occurred after these periods of fixation would not in some cases have preceded the arrival of the ball at the meter stick, these gaze shifts were not characterized as predictive gaze shifts.

All of the pitches for which a rapid gaze shift was originally found were initially examined. In the predictive condition, rapid gaze shifts occurred prior to or in the same video frame during which the ball struck the net, and then continued past the net in the direction of the meter stick for 25.0% of the pitches in which a rapid gaze shift occurred. For the other pitches in which a rapid gaze shift occurred (75.0%), the gaze lingered on the net at least briefly prior to shifting toward the meter stick. Two subjects demonstrated far more of these gaze shifts (11 gaze shifts) than the other subjects. In the swing condition, rapid gaze shifts occurred prior to or in the same video frame during which the ball struck the net, and then continued beyond the net toward the observer for 41.8% of the pitches in which a rapid gaze shift was originally found to occur. Once again, two subjects showed more of these gaze shifts (12 gaze shifts) than the other subjects. One of the subjects who executed the largest number of these gaze shifts in the predictive condition also executed the largest number of gaze shifts in the swing condition. Overall, there were 40 of these predictive gaze shifts in the predictive condition (mean = 2.67 ± 3.87, median = 0) and 49 in the swing condition (mean = 3.27 ± 4.43, median = 1). The number of these gaze shifts did not vary between the predictive and swing conditions (Wilcoxon signed rank test: median difference = −0.5, 95% confidence interval: (−2, 1), *p* = 0.388).

In a final analysis, the influence of baseball experience level on the difference in the total number of pitches with a rapid gaze shift for the predictive and swing conditions was evaluated. A two-way repeated measures analysis of variance was used. The experience level (college or high school) was included as a between-subject variable, and the condition (predictive or swing) was included as a within-subject variable. While the effect of condition was significant (F = 5.041, *p* = 0.043, η_p_^2^ = 0.279), the interaction term between experience level and condition was not significant (F = 0.116, *p* = 0.739, η_p_^2^ = 0.009), suggesting that experience level did not affect the relative number of rapid gaze shifts for the two conditions. The effect of experience level on the relative number of predictive gaze shifts beyond the net for the two conditions (predictive and swing) was also examined, once again using a two-way repeated measures analysis of variance. The condition factor (predictive or swing) was not significant (F = 0.188, *p* = 0.671, η_p_^2^ = 0.014) nor was the interaction term between experience level and condition (F = 0.030, *p* = 0.865, η_p_^2^ = 0.002).

#### 2.1.3. Discussion—Study 1

In this study, the swing condition was considered the coupled task, as this requires both an interpretation of the ball’s trajectory and a motor movement associated with swinging the bat. The predictive condition in which the subject estimated the ball’s passing height was the uncoupled task. There were subtle differences in the nature of the gaze movements between the 2 conditions. Specifically, the mean (and median) number of rapid gaze shifts was larger in the predictive condition compared to the swing condition. This difference did not, however, result from differences in the number of predictive gaze shifts. Instead, the most parsimonious explanation for the overall difference in the number of rapid gaze shifts between the predictive and swing conditions is that there were more rapid gaze shifts in the predictive condition prior to the time at which the ball arrived in the net.

One potential explanation for these differences is that foveal tracking may have been prioritized to a greater extent in the coupled swing task, perhaps because accurate retinal and extraretinal information is important for interceptive tasks [24]. On the other hand, in the uncoupled passing height judgment task, subjects may have prioritized the height information at the time the ball was stopped by the net to the same or to a greater extent than trajectory information obtained by minimizing rapid gaze shifts in tracking the ball. In that case, these rapid gaze shifts may have ensured fixation on the ball at the time the ball struck the net. As described in the literature, predictive fixations following a predictive gaze shift may serve to improve interceptive responses [21,22]. While those fixations on the ball at the net were generally not preceded by predictive gaze shifts, they might still be considered “predictive” fixations, in that the location of the ball at the net might facilitate predictions of ball location beyond the net in the direction of the meter stick or in the direction of bat–ball contact [21,22]. The idea that continuous tracking is prioritized more in the swing condition than in the predictive condition may be contradicted by the fact that there were 5 subjects who demonstrated poor tracking in the swing condition, compared to only 2 such subjects in the predictive condition. To fully assess those gaze tracking behaviors that account for differences in the number of rapid gaze shifts for the predictive and swing conditions, future studies in which gaze tracking and gaze tracking errors are assessed throughout the pitch trajectory will be required [28,29].

The lack of rapid gaze shifts in the direction of the plate in the predictive condition was unexpected, as was the finding that rapid gaze shifts in the direction of expected bat–ball contact in the swing condition were as common as the rapid gaze shifts in the direction of the plate in the predictive condition. It was thought that these predictive gaze shifts would occur more commonly in the uncoupled predictive condition. This hypothesis was based on the notion that in the predictive condition, subjects must compare the extrapolated location of the ball at the plate to the meter stick placed adjacent to the plate. To make this comparison efficiently, subjects would need to maintain trajectory information, gained from viewing and perhaps tracking the ball early in the trajectory, in working memory [25,26]. Trajectory information might be most effectively maintained in working memory by making a (rapid) predictive gaze shift toward the plate, which minimizes the time that trajectory information would need to be held in working memory. On the other hand, because continuous ocular tracking is known to contribute to interceptive tasks and because motor planning related to the bat swing must occur early in the pitch trajectory, continuous tracking was expected in the swing condition [2,24].

### 2.2. Study 2

An unexpected finding in Study 1 was that in both the swing and predictive conditions, subjects often stopped tracking the ball with their eyes at least briefly when the ball struck the net in front of them. This suggested a number of possibilities. First, it could be that subjects simply judge the passing height of an approaching ball based on the location of the ball when it stops moving. Alternatively, subjects may have stopped tracking the ball at the net because when the ball contacts the net, it briefly remains at that contact location, thereby providing unintended information by which to judge the ball’s height. Finally, it may be that gaze tracking of the ball stops at the net because observers believe there is no useful information to be gained at near distances, or because at near distances the angular velocity of the ball exceeds the capacity of gaze to track the ball [30,31]. For example, at the highest ball speeds utilized in this experiment, when the ball arrived at a distance of about 10 feet from the plate, the angular velocity of the ball exceeded about 80 deg/s. This angular velocity approaches or even exceeds the velocity limit for good ocular smooth pursuit performance [30,31]. There were two purposes of the second study. The first purpose was to determine whether, when passing height judgments were required, subjects would stop tracking pitched balls with their gaze at 8 feet (2.44 m) when there was no net to stop the ball from arriving at the observer. The second purpose was to determine whether ocular tracking was possible at near distances.

#### 2.2.1. Materials and Methods—Study 2

The protocol and the associated consent forms for the second study were also approved by the Ohio State University Biomedical Sciences Institutional Review Board. As in Study 1, subjects signed an informed consent form (IRB protocol #2024H0152) prior to participation in the study. Subjects were once again recruited using the Study Search website administered through The Ohio State University Center for Clinical and Translational Science, as well as through an e-mailed study advertisement to The Ohio State University College of Optometry staff, faculty, and students.

The same entrance criteria were used as in Study 1. Ten subjects (5 females) participated in the second experiment (mean age = 23.8, range 19 to 27). Four of these ten subjects had previously participated in Study 1. The subjects completed the same survey as that used in Study 1. Based on the survey responses, all of the females played softball while all of the males played baseball. All of the subjects were either primarily batters or both a pitcher and a batter, except for one subject who reported that they were primarily a pitcher. Excluding recreational play in college, the highest level at which 6 subjects played was the high school level. The other 4 subjects reported that they played at the collegiate level.

The same pitching machine and netting as that of Study 1 was used in Study 2. In all conditions, the Pupil Labs Pupil Core eye tracker was used to track the subject’s gaze relative to the scene viewed by the subject. While the eye tracker (eye-in-head rotation) sampling rate remained at 120 Hz, the sampling rate of the scene camera was changed to 60 Hz.

There were some differences in the procedures for Study 2 compared to those of Study 1. The plate and the subject were both about 20 inches (0.51 m) closer to the pitching machine in Study 2 so that the net could be placed behind the subject for one of the conditions. Further, three pitch speed settings were used in Study 2. Two of these speed settings (the slowest and the fastest speeds) were the same as those used in Study 1, and the third was between these two original speeds. The speed of the balls at each of these 3 speed settings was checked at a point as close as possible to the time at which the ball exited the pitching machine tube using a radar device (Ball Coach Radar, Pocket Radar Inc., Santa Rosa, CA, USA). Five pitches were assessed at each speed setting. Mean measured speeds (and standard deviations) were 84.0 ± 0.71 mph (135.18 km/h) (fast setting), 77.4 ± 1.52 mph (124.56 km/h) (medium setting), and 67.8 ± 0.45 mph (109.11 km/h) (slow setting). Height measurements were once again performed at each speed setting in a similar manner as described in Study 1. However, these height measurements were only made at the plate. The mean (and standard deviations) of these heights (10 pitches at each speed setting) were 97.27 ± 3.69 cm (fast setting), 86.67 ± 5.23 cm (medium setting), and 63.67 ± 3.91 cm (slow setting). Subjects viewed 30 pitches in each of 3 randomized conditions (90 pitches total). Each of the three speed settings was used 10 times in each condition, except in the case of one subject where for two pitches the speed was accidentally set at a lower-than-intended value.

The first two conditions were randomized for each subject. In both conditions, the subject was asked to estimate the passing height of pitches using a two-meter ruler placed in the batter’s box opposite the subject. For one of these conditions, the net was placed about 8 feet (2.44 m) in front of the subject as in Study 1. For the other condition, the net was moved to a location just behind the subject so that the pitched balls passed by the subject. The third condition was completed last for all subjects. In this condition the net was placed behind the subject, the meter stick was removed, and the subject was asked to “track the ball all the way in with their gaze”. This final condition was included to assess whether the subject was able to track the ball continuously over at least a portion of the final 8 feet (2.44 m) of the ball’s trajectory.

For each of the ten subjects, 20 pitches were analyzed for each of the three conditions, such that a total of 600 pitches were analyzed. One experimenter (M.C.) assessed gaze movements in all of the videos. This investigator viewed each pitch as recorded from the scene camera frame-by-frame at 60 Hz in the Pupil Player software.

#### 2.2.2. Results—Study 2

The first 20 pitches of each of the three conditions were analyzed for 9 subjects. For the remaining subject, 20 pitches for each condition were still analyzed, but it was not the first 20 due to maladjustment of the pitch speed control. Similar criteria to those of Study 1 were used to classify rapid gaze shifts as predictive or tracking movements in Study 2. That is, rapid gaze shifts were considered predictive if they extended past the ball and if they were initiated prior to or at the time that the ball struck the net. Rapid gaze shifts were considered tracking movements if they started behind the ball and ended at the approximate position of the ball.

In all 3 conditions, for most subjects gaze tracking of the ball usually continued until near the time the ball struck the net regardless of whether the net was in front of or behind the plate. Rapid gaze shifts occurred in 33.7% of the trials. Most of these gaze shifts were tracking movements (81.2% of the total gaze shifts) rather than predictive gaze shifts (18.8% of the total rapid gaze shifts). The mean (and standard deviation) for the number of rapid gaze shifts for each condition was as follows: height prediction with net in front of subject (8ft) = 10.5 ± 7.7, height prediction with net behind subject = 7.0 ± 5.2, tracking condition = 2.7 ± 2.9. There was a significant difference between the number of rapid gaze shifts for these conditions (Friedman: Chi square = 9.632, degrees of freedom = 2, *p* = 0.008). The Wilcoxon signed rank test was used to compare the number of rapid gaze shifts for all conditions. The Bonferroni correction was used to adjust the significance level for these three comparisons (α = 0.017). The results of these comparisons were (1) height prediction with net in front versus height predication with net behind: Z = −2.02, *p* = 0.043 (2) height prediction with net behind versus tracking: Z = −1.94, *p* = 0.052 (3) height prediction with net in front versus tracking: Z = −2.31, *p* = 0.021. None of these comparisons were significant (*p* > 0.017 in all cases) although the difference in the number of rapid gaze shifts just missed significance for the height prediction in front and tracking conditions.

Comparing the conditions where the net was in front of the subject to the condition where the net was behind the subject, one subject made an equal number of rapid gaze shifts, one subject made more rapid gaze shifts with the net behind, and the other eight subjects made more rapid gaze shifts when the net was in front of them. This result was surprising, since fewer rapid gaze shifts were expected over the shorter trajectory when the net was in front of the subject. Further work will be required to assess the implication of this latter finding.

It was also of interest to examine the passing height responses when the net was in front of the subject and when the net was behind the subject. These responses reflect on whether subjects continue to update their passing height estimates when the ball approaches within 8ft (2.44 m). Only 9 subjects were included in this analysis, because as mentioned before, for one subject there were errors in the speed settings that precluded proper correlation of height responses to pitch speed. The results are shown in Table 2 below, where the mean height responses (mean of subject means) when the net was in front of and behind the subject are plotted against the pitch speed. Also included in this table are the measured heights of the ball at the plate for each speed setting, and the visually estimated heights of the ball at the net when the net was behind the plate.

From Table 2, the subjects’ passing height responses were too high when the net was 8 feet (2.44 m) in front of the subjects and too low when the net was behind the subjects. Subject responses when the net was behind the subjects were close to those expected if judgements were made based on where the ball struck the net. A two-way repeated measures analysis of variance was completed for those data in Table 2. The responses were the subject passing height estimates and the factors were net location (in front or behind the subject) and pitch speed. The Greenhouse–Geisser correction was applied to the results because Mauchly’s test of sphericity was significant (*p* < 0.05) for both the pitch speed variable and the interaction term. The results are shown in Table 3. Both the pitch speed and the net location variables were significant (*p* < 0.001), as was the interaction term (*p* = 0.007). Because the interaction term was significant, a paired t-test was performed to compare the height estimates for the two net locations at each speed. All three of these comparisons were significant when the significance level was adjusted using the Bonferroni correction (*p* < 0.017).

#### 2.2.3. Discussion—Study 2

The results of Study 1 raised concerns that the net in front of the subject had become an unintended variable since subjects commonly stopped their gaze at least briefly near the location where the ball contacted the net. This could suggest that subjects simply judge the passing height of an approaching ball based on the location of the ball when it stops moving. Alternatively, it was possible that subjects may have stopped tracking the ball when it contacted the net not because the ball stopped moving at that point, but because when the ball strikes the net, it briefly lingers at the location where it contacts the net and then drops to the ground. Fixation near the location where the ball contacts the net could provide trajectory information that could facilitate passing height judgments [21,22]. Alternatively, it may be that ocular ball tracking stops at the net because observers believe there is no useful information on passing height to be gained at near distances (or they do not use the information at near distances), or because at near distances the angular velocity of the ball exceeds the capacity of the eyes to track the ball.

First, the fact that subjects were generally able to maintain fixation on or near the ball at distances closer than 8 feet (2.44 m) suggests that the angular velocity of the ball at 8 feet (2.44 m) did not exceed the subject’s ability to maintain gaze on the ball. Second, the passing height judgments were clearly influenced by the location of the net. That is, when the net was placed behind the subject, the height judgments were lower at all speeds compared to those when the net was placed in front of the subject. One possibility for these results is that subjects based their passing height judgments on the latest visual information available during the pitch [32]. That is to say, the height of the ball when it stops moving is the passing height estimate with no further trajectory extrapolation. On the other hand, it may be that trajectory extrapolation is ongoing or continuous throughout the pitch and cues for trajectory estimation are better when the ball reaches near distances. If in fact subjects are making their passing height estimates based on the ball’s location at the time it strikes the net, there is another question that remains unanswered. This question is whether passing height judgments are based on information related to the location of the ball as it lingers on the net, or whether these judgments are based on the final location of the ball when it stops moving. While these possibilities cannot be conclusively differentiated from one another, a previous study by Benson and colleagues showed that passing height judgments were like those in the current study (i.e., too high) when the view of an approaching ball was blocked with occlusion spectacles [33]. This suggests that the location of the ball as it lingers on the net may be less important in informing passing height estimates than the final height at which the ball is seen.

In Study 2, there was also no significant difference in the number of rapid gaze shifts for any of the conditions. However, the difference in the number of rapid gaze shifts just missed significance for the height estimation task with the net in front of the subject condition and the condition where the subject was asked to track the ball all the way to the net behind the subject. In this latter comparison, the mean number of rapid gaze shifts was greater in the condition where the net was in front of the subject. These results might reflect a transition between reliance on rapid tracking gaze shifts in the condition where the net was in front of the subject to reliance on gaze pursuit tracking in the “track to the net behind” condition. As was stated in the discussion in Study 1, future studies assessing gaze tracking and gaze tracking errors throughout the pitch trajectory will be required to address the possibility that gaze pursuit tracking was more common in the “track to the net behind” condition [28,29].

## 3. Study Limitations

There are some limitations in this study. As mentioned before, the coupled task was only partially representative of the task in actual games. The extent to which eye movements and visuomotor behaviors vary between coupled and uncoupled tasks has not yet been fully resolved, so it is unclear how a greater degree of coupling in the swing condition may have affected the results [2,4,5,33]. The other limitation of this study is that because quantitative measures of head movements were not obtained, the gaze shifts and rapid gaze shifts in this study were not subdivided into eye movements only, head movements only, and combined eye and head movements. Further, eye movements were not subdivided into different eye movement types including smooth pursuit, catch-up saccades, and predictive saccades [21,34,35,36]. In future studies, an examination of those eye and head movements that make up the gaze shifts will provide more details of how eye and head movements vary in coupled and uncoupled tasks.

## 4. Conclusions

In conclusion, the results of Study 1 demonstrated that the difference in the number of predictive gaze shifts in the direction of the meter stick in the predictive condition and in the direction of expected bat–ball contact in the swing condition were not significantly different. This could suggest that in coupled and uncoupled conditions, similar numbers of predictive gaze movements occur in response to approaching balls. This result contrasts with that of Dicks and colleagues [6]. These investigators demonstrated that football (soccer) goalkeepers tended to look at the opposing player’s movements in facing penalty kicks when the task was uncoupled (verbal indication of the direction of the penalty kick or a “side step” and arm placement in the anticipated kick direction) compared to a coupled task (attempting to intercept or “save” the kick). Methodological differences between the current study and that of Dicks and colleagues could account for the disparate results between these studies. In the current study, there was no pitcher throwing the ball, so the location of fixations and perhaps the gaze movements at least early on may have varied in the manner suggested by Dicks and colleagues had a pitcher been present [37]. Additionally, the coupled task in the study of Dicks and colleagues was likely more representative of a goalkeeper’s task in competitions compared to that in the current study. The extent to which this provides an explanation of the disparate results of Dicks and colleagues and those in the current study is unknown, because the degree of coupling necessary to obtain behaviors like those in competitions is unclear [38]. Finally, the study by Dicks and colleagues examined ocular fixations, unlike the current study, which examined gaze movements in response to an approaching ball. Perhaps differences in ocular fixations for coupled and uncoupled tasks are greater than differences in gaze movements in these tasks.

While predictive gaze tracking movements were similar between the coupled and uncoupled conditions, there were subtle differences in gaze behavior in the coupled (partial swing) and uncoupled (passing height judgment) conditions. Specifically, the mean number of rapid gaze shifts was greater in the uncoupled condition. This may suggest that foveal gaze tracking is prioritized to a greater extent in the coupled condition, but future studies in which gaze tracking and gaze tracking errors are evaluated throughout the pitch will be necessary to determine how gaze tracking strategies vary in coupled and uncoupled conditions.

In Study 2, the possibility that subjects simply chose to make passing height judgments at 8 feet (2.44 m) in front of the plate, or the possibility that subjects could not track the ball with their gaze when the ball was within 8 feet (2.44 m) of the plate were examined. Subjects were able to track the ball at distances closer than 8ft from the plate. In addition, passing height judgments varied between the condition where the ball was stopped at 8 feet in front of the subject and the condition where the ball was stopped behind the subject. Taken together, these results suggest that passing height judgments are continuously evaluated throughout the ball’s flight.

There are potential variables that were not included in this study, but their effect on gaze tracking in batting could be addressed in future studies. These variables include the predictability of pitch trajectories and the extent to which actual batting of the ball is allowed. Finally, responses may vary in subjects with higher levels of experience.

## Figures and Tables

**Table 1:** Mean ball speed and standard deviation in miles per hour (kilometers per hour in parentheses) based on the time required for the ball to traverse incremental distances from the pitching machine.

Speed	10 Foot (0.30 m) Distance	20 Foot (6.10 m) Distance	30 Foot (9.14 m) Distance	40 Foot (12.19 m) Distance
Higher	83.07 ± 1.50 (133.69)	80.59 ± 1.20 (129.70)	78.74 ± 1.43 (126.72)	76.75 ± 1.59 (123.52)
Lower	65.82 ± 1.55 (105.93)	64.16 ± 1.00 (103.26)	62.71 ± 0.98 (100.92)	61.35 ± 0.91 (98.73)

**Table 2:** Subject passing height estimates (mean of subject means and standard deviation) when the net was 8 feet (2.44 m) in front of the subjects and when the net was behind the subjects at each pitch speed. Included are measurements of the actual height of the balls at the plate (mean and standard deviation) and visual estimates of the height of the balls at the net when the net was placed behind the subjects.

Speed	Height Prediction—Net in Front (cm)	Height Prediction—Net Behind (cm)	Measured Height at the Plate (cm)	Estimated Height at the Net Behind Subjects (cm)
67.8 mph (109.11 km/h)	79.99 ± 14.75	49.37 ± 9.65	63.67 ± 3.91	50
77.4 mph (124.56 km/h)	95.03 ± 14.2	68.69 ± 7.09	86.67 ± 5.23	77
84.0 mph (135.18 km/h)	105.26 ± 13.67	83.93 ± 7.11	97.27 ± 3.69	91

**Table 3:** Results of the two-way repeated measures analysis of variance for the height estimates in Study 2.

Factor	F Statistic	*p*-Value	Partial Eta Squared (η_p_^2^)
Speed	147.421	*p* < 0.001	0.949
Net location	25.686	*p* < 0.001	0.763
Interaction term (speed*net location)	10.246	*p* = 0.007	0.562

## Data Availability

The original contributions presented in the study are included in the article. Further inquiries can be directed to the corresponding author.
